# Genome-Wide Testing of Putative Functional Exonic Variants in Relationship with Breast and Prostate Cancer Risk in a Multiethnic Population

**DOI:** 10.1371/journal.pgen.1003419

**Published:** 2013-03-28

**Authors:** Christopher A. Haiman, Ying Han, Ye Feng, Lucy Xia, Chris Hsu, Xin Sheng, Loreall C. Pooler, Yesha Patel, Laurence N. Kolonel, Erin Carter, Karen Park, Loic Le Marchand, David Van Den Berg, Brian E. Henderson, Daniel O. Stram

**Affiliations:** 1 Department of Preventive Medicine, Keck School of Medicine and Norris Comprehensive Cancer Center, University of Southern California, Los Angeles, California, United States of America; 2 Epidemiology Program, Cancer Research Center, University of Hawaii, Honolulu, Hawaii, United States of America; University of Washington, United States of America

## Abstract

Rare variation in protein coding sequence is poorly captured by GWAS arrays and has been hypothesized to contribute to disease heritability. Using the Illumina HumanExome SNP array, we successfully genotyped 191,032 common and rare non-synonymous, splice site, or nonsense variants in a multiethnic sample of 2,984 breast cancer cases, 4,376 prostate cancer cases, and 7,545 controls. In breast cancer, the strongest associations included either SNPs in or gene burden scores for genes *LDLRAD1*, *SLC19A1*, *FGFBP3*, *CASP5*, *MMAB*, *SLC16A6*, and *INS-IGF2*. In prostate cancer, one of the most associated SNPs was in the gene *GPRC6A* (rs2274911, *Pro91Ser*, OR = 0.88, P = 1.3×10^−5^) near to a known risk locus for prostate cancer; other suggestive associations were noted in genes such as *F13A1*, *ANXA4*, *MANSC1*, and *GP6.* For both breast and prostate cancer, several of the most significant associations involving SNPs or gene burden scores (sum of minor alleles) were noted in genes previously reported to be associated with a cancer-related phenotype. However, only one of the associations (rs145889899 in *LDLRAD1*, p = 2.5×10^−7^ only seen in African Americans) for overall breast or prostate cancer risk was statistically significant after correcting for multiple comparisons. In addition to breast and prostate cancer, other cancer-related traits were examined (body mass index, PSA level, and alcohol drinking) with a number of known and potentially novel associations described. In general, these findings do not support there being many protein coding variants of moderate to high risk for breast and prostate cancer with odds ratios over a range that is probably required for protein coding variation to play a truly outstanding role in risk heritability. Very large sample sizes will be required to better define the role of rare and less penetrant coding variation in prostate and breast cancer disease genetics.

## Author Summary

For breast and prostate cancer, GWAS have revealed many risk variants (>70 for each cancer as of this report). All together the common variants in these regions explain only a minority of familial risk of these cancers. Using the Illumina HumanExome SNP array, we explored the hypothesis of rare coding variation contributing to breast and prostate cancer risk in a sample of African American, Latino, Japanese, Native Hawaiian, and European American breast and prostate cancer cases and controls from the Multiethnic Cohort study. While only one association exceeded significance thresholds after correcting for multiple comparisons, a number of suggestive associations involving genes previously reported to be associated with a cancer-related phenotype were noted. Our results do not generally support a major role of protein-coding variants with odds ratios over a range that is probably required for protein coding variation to play a truly outstanding role in risk heritability. If very rare and/or less penetrant coding variants underlie disease heritability of these cancers, then very large sample sizes (i.e. consortia) will be required for their discovery.

## Introduction

For most common diseases and traits the genetic basis underlying susceptibility has yet to be completely revealed. While genome-wide association studies (GWAS) have been remarkably successful in identifying common genetic variants associated with risk, the effect sizes of the risk alleles have been modest (relative risk, RR of 1.1–1.4) and in most cases, even in sum, they can explain only a fraction of familial risk or disease heritability. GWAS have relied almost exclusively on Illumina and Affymetrix SNP arrays, with SNP content selected primarily from HapMap to capture a large fraction of common variation in coding and non-coding regions in populations of European ancestry. The vast majority of alleles with frequencies <5%, and in particularly those with frequencies ≤1%, have not been tested. This low allele frequency spectrum of genetic variation represents a very large fraction of all variation in the human genome. Thus, to date, a large fraction of genetic variation has yet to be explored with respect to disease etiology.

It is possible that the majority of less common (1–5%) and rare variants (<1%) will have weak effects, like the GWAS-identified common variants, and if this is the case then very large studies will be required for their discovery. An alternative hypothesis is that less common and rare variants convey larger relative risks than common variants, and indeed this assumption is required in order that rare variants contribute meaningfully to the understanding of inherited susceptibility. Such enhancement of effect sizes for rarer alleles may be especially relevant to rare coding variants given their dominant role in the etiology of “Mendelian” disorders (e.g. the OMIM database [1]). Support for the hypothesis that rare coding variation also profoundly affects risk of certain “complex” diseases is growing and there are now a number of such examples including rare missense variants in *CHEK2*, *ATM*, *NBS1*, *RAD50*, *BRIP1*, and *PALB2* in breast cancer [2], rare coding mutations in *RAD51D* and *BRIP1* in ovarian cancer [3], [4], as well as rare coding variants in genes implicated in hyperglyceridemina [5] and colorectal cancer adenomas [6]. More recently, whole-genome and candidate gene sequencing studies have revealed rare coding variants in *ALDH16A1* for gout [7] and a number of genes (*NOD2*, *IL23R*, *CARD9*, *IL18RAP*, *CUL2*, *C1orf106*, *PTPN22* and *MEC19*) involved in inflammatory bowel disease [8]. Studies in prostate cancer have reported rare gene coding mutations in *BRCA2* (found in 2% of cases <55 years) to be associated with greater risk of prostate cancer (RR>4.5) and more aggressive disease [9], [10]. For many of these examples, in addition to single SNP association testing, burden of rare variation analyses have been applied to increase the number of observations in the comparison groups (and thus the statistical power), and to provide statistical support for the involvement of the gene which is not achieved when examining large number of SNPs in any given gene.

To date, a lack of technology to survey the genome and accurately enumerate and test the variants in large numbers of samples has limited the exploration of less common and rare alleles. In the past year the Illumina Infinium HumanExome array (or “exome chip”) has been developed in collaboration with investigators who combined whole-exome sequencing conducted in >12,000 individuals of primarily European ancestry as well as in small numbers of other racial/ethnic minorities including African Americans, Hispanics, and Asians; the content on the array includes >200,000 putative functional exonic variants and is aimed to provide comprehensive testing on all non-synonymous variants above 0.1% frequency in Europeans. In the present study, we have utilized this array to test the hypothesis that there are less common and rare functional variants in the coding regions of genes that convey risk for breast and prostate cancer of greater magnitude than the common variants revealed through GWAS. We tested both single markers as well as gene summaries of the burden of rare alleles in multiethnic studies of invasive incident breast cancer and prostate cancer in the Multiethnic Cohort study (MEC: 3,141 breast cancer cases, 4,675 incident prostate cancer cases and 8,021 controls). In addition we conducted exploratory analyses of rare variants in relationship with several breast and prostate cancer-related traits ascertained at baseline in the entire MEC sample (n = 15,837).

## Results

**Figure 1:**
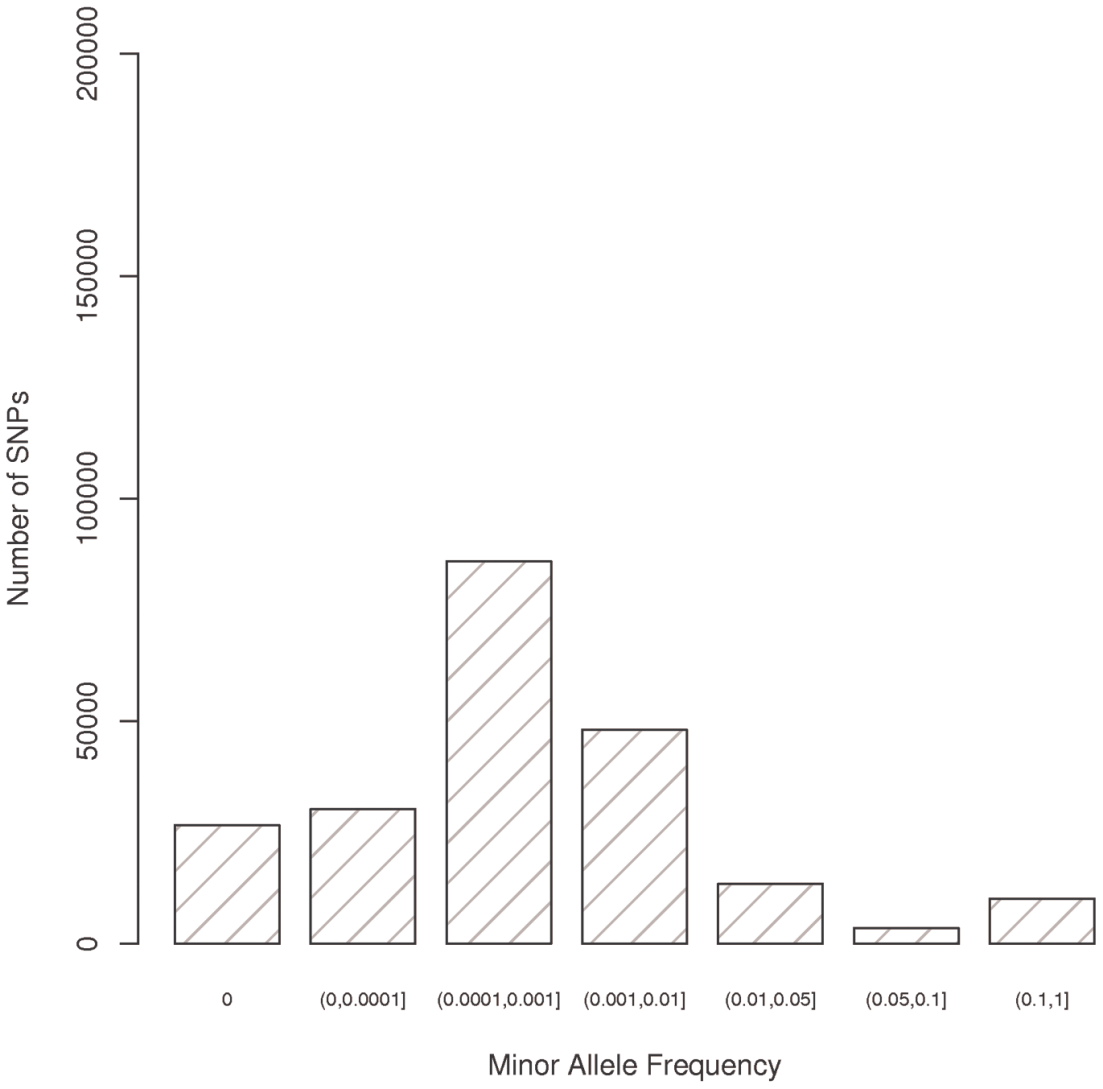
Minor allele frequency for all variants successfully genotyped using the Illumina Human Exome array.

**Figure 2:**
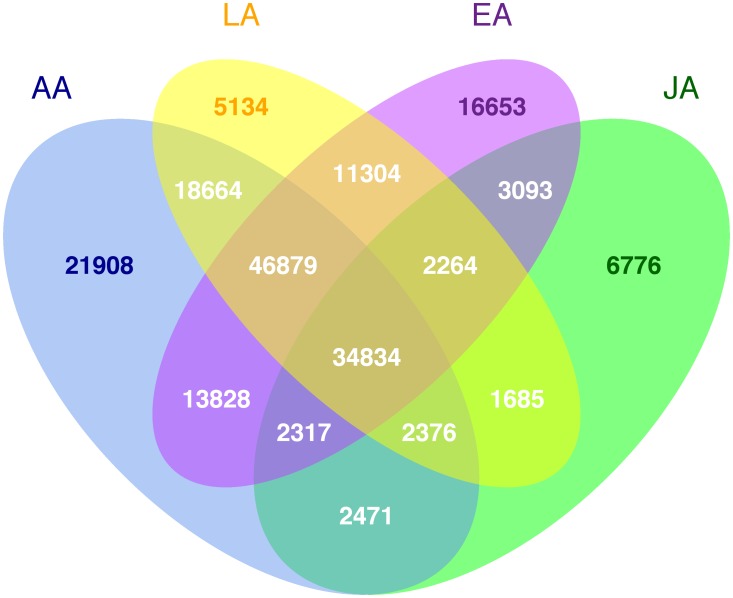
Number of polymorphic putative functional variants by racial/ethnic group.

The analysis included 217,601 putative functional variants (of 247,870 total markers listed on the array), predicted to alter the protein coding sequence, and which passed quality control procedures (see Methods). Of the 15,837 samples, 14,905 were included in the analysis (3,315 European Americans, 3,854 African Americans, 3,106 Latinos, 3,843 Japanese Americans and 787 Native Hawaiians; see Methods for exclusion criteria). A few mitochondrial SNPs were included on the array (n = 165 SNPs passing quality control) but are not discussed here (no associations with them were seen in the top ranked 1,000 associations for either breast or prostate cancer). The number of breast and prostate cancer cases and controls are shown in Table 1. In this multiethnic sample, 191,032 (88%) putative functional variants were found to be polymorphic in at least one population, with 26,569 (12%) being monomorphic in all five populations (Figure 1). The percentage of monomorphic SNPs ranged from 34.1% in African Americans, 39.6% in European Americans and 43.3% in Latinos to 66.8% in Native Hawaiians and 74.2% in Japanese Americans (Figure S1). Of the polymorphic SNPs, 178,776 (93.4%) were nonsynonymous (NS) variants, 8,308 (4.4%) splice site (SP) variants, and 3,948 (2.1%) nonsense variants which either lead to a gain or loss of a stop codon. Of the polymorphic SNPs, 34,834 (18.2%) were polymorphic in all four of the largest populations (excluding Native Hawaiians), with 81,713 SNPs (42.7%) being polymorphic in African Americans, Latinos and European Americans (Figure 2). African Americans had the largest number of unique polymorphic SNPs (21,908, 11.4%), followed by European Americans (16,653, 8.7%), Japanese Americans (6,776, 3.5%) and Latinos (5,134, 2.7%).

**Table 1:** The Descriptive Characteristics of the Multiethnic Case-Control Studies of Breast and Prostate Cancer.

Breast Cancer	n (Cases/Controls)	Age (mean(years)[sd]; Cases/Controls)	n ER+/n ER − (n (%))
All Groups	2984/7545	67[8.8]/68[8.6]	1688(56.6)/441(14.8)
European Americans	754/1682	66[8.8]/68[8.9]	450(59.7)/95(12.6)
African Americans	591/2146	68[9.3]/69[8.4]	311(52.6)/130(22.0)
Latinos	614/1302	67[8.2]/67[7.8]	339(55.2)/112(18.2)
Japanese Americans	809/2012	66[8.6]/69[8.6]	467(57.7)/84(10.4)
Native Hawaiians	216/403	64[8.3]/64[8.6]	121(56.0)/20(9.3)

Prostate Cancer	n (Cases/Controls)	Age (mean(years)[sd]; Cases/Controls)	n Advanced/n Non-advanced (n (%))
All Groups	4376/7545	70[7.2]/68[8.6]	499(11)/3666(84)
European Americans	879/1682	69[7.7]/68[8.9]	100(11)/749(85)
African Americans	1117/2146	70[7.3]/69[8.4]	116(10)/932(83)
Latinos	1190/1302	69[6.6]/67[7.8]	145(12)/986(83)
Japanese Americans	1022/2012	72[7.4]/69[8.6]	114(11)/863(84)
Native Hawaiians	168/403	69[6.7]/64[8.6]	24(14)/136(81)

In the pooled sample, 190,662 putative functional (NS, SP, or stop) SNPs had a minor allele frequency (MAF) <1% (56,759<0.01%; 85,897 between 0.01% and 0.1%, and 48,006 between 0.1% and 1%) (Figure 1, Figure S1). The minor allele frequency distributions were similar across three of the five populations with African Americans, European Americans and Latinos having roughly the same number of SNPs with frequencies greater than 0 and less than 1% (100–110 thousand); However there were only 37,979 SNPs with a frequency above zero and less than 1% in Japanese Americans and 52,985 in Native Hawaiians. The number of SNPs with a frequency >1% ranged from approximately 18–35 thousand between sampled populations.

Inspection of the distribution of the chi-square (score) tests from models for overall breast or prostate cancer showed evidence of over-dispersion of test statistics (genomic control lambda estimate to be approximately 1.15 for breast and 1.20 for prostate) however when very rare SNPs were removed (MAF<0.1% overall) then the Wald statistics appeared to be sampled from an overall central chi-square distribution (genomic control lambda = 1.00 for breast cancer and lambda = 1.05 for prostate cancer). In the gene burden analyses, the distribution of observed score tests showed mild evidence of over-dispersion (lambda = 1.04 for breast cancer and lambda = 1.06 for prostate cancer). When the single SNP analysis was restricted to estrogen receptor-negative (ER-) breast or advanced prostate cancer, where there were many more controls than cases included in each model, then the behavior of the score test for the single SNP associations was problematic for rare SNPs. For such SNPs we followed up any apparently globally significant associations with exact logistic regression analysis, in order to reduce what appeared to be a proliferation of false positive signals.

The total number of genes having at least one polymorphic functional variant genotyped and passed quality control varied slightly between breast (17,168 genes) and prostate cancer (17,203 genes) due to sampling (i.e. some variants were polymorphic only for breast cancer cases and so were not included in the prostate cancer analyses and vice versa).

### Breast Cancer Single SNP Associations

In the ethnic-pooled breast cancer analyses (2,984 cases and 7,545 controls), the most significant predicted protein-altering variant was a rare SP variant rs145889899 at the splice donor site in the second intron of the gene *LDLRAD1* (OR = 3.74, p = 2.5×10^−7^), which was almost exclusively seen in African Americans, this variant was statistically significant at our exome-wide level (nominal p<3.9×10^−7^, see Methods). Of the top 10 ranked associations, the remaining 9 involved NS variants (p-values ≥1.3×10^−6^, Table 2, Table S1). None of the other associations met the Bonferroni adjustment for multiple comparison testing. All of the 10 most associated variants, were quite rare and present mainly or exclusively in one or two ethnic groups. The genes containing the most significant SNPs for breast cancer ranged widely in apparent function (see Table 2) with GWAS associations reported with SNPs in *CFB* (complement factor B) for age-related macular degeneration [11], *BAZ2A* for platelet counts [12] and *ACADS* for metabolic traits [13].Table S1 gives information for the 100 most significant associations for breast cancer, both overall and by ethnic group when including all SNPs passing quality control (not just the non-synonymous, splice site and nonsense variants described here).

**Table 2:** The Most Significant Associations of Single Coding Variants with Breast Cancer Risk.

All Cases (n = 2,984) vs Controls (n = 7,545)													
SNP ID^a^	Chr	Position^b^	rs#	A1/A2^c^	Type	Gene	OR^d^	P	AAMAF^e^	NHMAF	JAMAF	LAMAF	EAMAF
exm61019	1	54476084	rs145889899	T/C	*SP*	*LDLRAD1*	3.74	2.50E-07	0.0065	0	0	0	0
exm1579798	21	46950811	rs142899279	A/C	*Val342Phe*	*SLC19A1*	12.67	1.30E-06	0	0.0025	0	0	0.00030
exm841657	10	93668692	NA	A/G	*Pro12Leu*	*FGFBP3*	>>	2.50E-06	0	0	0	0	0
exm952402	11	104878019	rs45585331	C/A	*Leu17Arg*	*CASP5*	9.69	2.50E-06	0.00093	0	0	0	0
exm533277	6	31918154	rs149101394	G/A	*Lys533Arg*	*CFB*	2.90	3.50E-06	0.0075	0.0012	0.00025	0.00077	0.00089
exm510328	5	179285781	NA	A/G	*Arg6Cys*	*C5orf45*	11.61	4.20E-06	0.00070	0	0	0	0
exm1043849	12	121177159	rs28940872	T/C	*Arg383Cys*	*ACADS*	25.69	7.10E-06	0.00023	0	0	0	0
exm1013941	12	56998559	NA	T/C	*Val927Met*	*BAZ2A*	>>	1.30E-05	0	0	0	0	0
exm1234521	16	30795481	NA	G/C	*Lys56Asn*	*ZNF629*	>>	1.30E-05	0	0	0	0	0
exm132287	1	186313108	rs58030082	T/C	*Val1178Ile*	*TPR*	1.59	1.30E-05	0	0.021	0.056	0.00038	0

ER+ Cases (n = 1,688) vs Controls (n = 7,545)													
SNP ID^a^	Chr	Position^b^	rs#	A1/A2^c^	Type	Gene	OR^d^	P	AAMAF^e^	NHMAF	JAMAF	LAMAF	EAMAF
exm1573155	21	43529776	NA	A/G	*Ala542Thr*	*UMODL1*	7.28	9.80E-07	0.0021	0	0	0	0
exm85453	1	114380886	rs138092829	C/T	*Asp379Gly*	*PTPN22*	12.12	1.60E-06	0.00093	0	0	0	0
exm1358833	17	74625633	rs115756441	T/C	*Gly98Arg*	*ST6GALNAC1*	3.26	2.40E-06	0.010	0	0	0.0012	0.00030
exm1159729	15	48734008	rs113577372	A/C	*Ala2025Ser*	*FBN1*	7.66	2.70E-06	0.0019	0	0	0	0
exm1093791	14	24792132	rs74387312	G/A	*Tyr774His*	*ADCY4*	13.00	2.90E-06	0.00023	0	0	0	0.00059
exm1621500	22	50682785	rs139896192	T/C	*Arg35Gln*	*TUBGCP6*	10.16	3.70E-06	0.0012	0	0	0	0
exm900686	11	36119939	rs147309219	G/A	*Lys128Glu*	*LDLRAD3*	11.10	3.80E-06	0.00093	0	0	0	0
exm551694	6	44274257	rs139372744	A/G	*Pro388Ser*	*AARS2*	4.71	4.20E-06	0.0044	0	0	0	0
exm178049	2	26418053	rs137852769	G/C	*Glu510Gln*	*HADHA*	11.73	5.20E-06	0.00047	0	0	0.00038	0
exm412038	4	88534411	NA	A/G	*Arg358Lys*	*DSPP*	9.39	9.30E-06	0.0012	0	0	0	0

ER- Cases (n = 441) vs Controls (n = 7,545)													
SNP ID^a^	Chr	Position^b^	rs#	A1/A2^c^	Type	Gene	OR^d^	P	AAMAF^e^	NHMAF	JAMAF	LAMAF	EAMAF
exm1165463	15	59009800	rs144893047	T/C	*Arg61His*	*ADAM10*	8.52	1.30E-09	0.0040	0	0	0	0
exm221867	2	113539232	rs142134831	T/G	*Gln90Lys*	*IL1A*	9.27	1.30E-08	0.0030	0	0	0.00038	0
exm220393	2	110959008	rs145479679	G/T	*Ile45Leu*	*NPHP1*	7.40	8.40E-08	0.0047	0	0	0	0.00030
exm61019	1	54476084	rs145889899	T/C	SP	*LDLRAD1*	6.17	1.40E-07	0.0065	0	0	0	0
exm645918	7	101183198	rs190166648	T/C	*Arg156Trp*	*EMID2*	14.51	1.50E-07	0.00070	0	0	0	0.00059
exm492809	5	147505341	NA	A/G	Arg962Lys	*SPINK5*	9.57	1.60E-07	0.0028	0	0	0	0
exm1253047	16	69727480	rs145602190	G/A	*Gln1157Arg*	*NFAT5*	5.39	1.70E-07	0.0021	0.0037	0	0.0035	0.0051
exm1292126	17	8273026	rs78738842	A/G	*Ala302Val*	*KRBA2*	6.42	2.70E-07	0	0	0.0092	0	0
exm741581	9	18928963	rs150639454	A/G	*Thr171Ile*	*FAM154A*	11.10	2.70E-07	0.0021	0	0	0	0
exm2276233	12	15734702	rs144347297	A/C	*Asp235Glu*	*PTPRO*	6.47	3.00E-07	0.0019	0	0	0.0050	0

a SNP ID from HG19.

b Position based on GRCh37.

c A1 is minor allele based on the entire multiethnic sample and the tested allele, A2 is the reference allele.

d Odds ratio per allele based on the pooled analysis adjusted for age and the first 10 principle components.

e MAF is minor allele frequency in controls.

AA, African Americans; NH, Native Hawaiians; JA, Japanese Americans; LA, Latinos; EA, European Americans; SP, splice-site variant.

For ER- breast cancer (n = 441 cases) many associations (358) with very rare SNPs were nominally significant using the score test but the p-values failed to stand up to further investigation using exact logistic regression (the exact p-values ranged from 3×10^−5^ to 0.21). The many small p-values apparently reflected overly liberal behavior of the score test when alleles are rare and when there are many more cases than controls. In order to reduce discussion of a large number of likely false positive tests we consider in the subtype analyses only SNPs with at least 10 minor alleles seen over all cases and controls. With this restriction we found a total of ten globally significant SNPs (using the score test). However, p-values from exact logistic regression for these SNPs were again far less striking (ranging from 3×10^−5^ to 1.5×10^−3^).

When restricted to estrogen receptor-positive (ER+) cases (n = 1,688) (and screening out SNPs with less than 10 minor alleles seen) the most significant coding SNP was a rare NS variant in *UMODL1* (exm1573155, *Ala542Thr*, OR = 7.28, p = 9.8×10^−7^) (Table 2, Table S2). This SNP had a frequency of just over 0.2% in African Americans controls and 0 in the other groups. No associations are reported for this gene in the GWAS catalog. Neither this SNP nor any others were significant after correction for multiple testing.

In ethnic-specific analyses of overall breast cancer only one additional SNP (in *FANCI*) met our criteria (p<3.9×10^−7^) of global significance. This NS variant (rs62020347, *Pro55Leu*) was common in European Americans, African Americans, and Latinos (3–8% frequency) but was only associated with risk among European Americans (MAF 8%, OR = 0.47, p = 1.8×10^−7^) and was weakly associated with risk overall (p = 0.02) (Table S1).

### Breast Cancer Gene Burden Analysis

Table 3 summarizes the most significant findings from the gene burden (sum of coding variants) analysis based on all common and rare (≤1%) functional SNPs in each gene. Further details are given in Table S5. For overall breast cancer no gene burden sum passed the Bonferroni criteria (3×10^−6^) for global significance for testing approximately 17,200 genes (see Methods). The strongest associations were seen for *MMAB* (p = 5.0×10^−5^), *SLC16A6* (p = 5.0×10^−5^) and *INS-IGF2* (p = 1.2×10^−4^). The *MMAB* gene is close to non-exonic SNPs that have been associated with HDL cholesterol [14] and one of those GWAS SNPs (the intronic variant rs7134594) was among our top 100 single SNP associations with breast cancer (Table S1). *INS-IGF2* contains an intronic SNP that has been associated with type 1 diabetes [15]. Restricting the gene burden analysis to only SNPs with overall frequency ≤1% gave non-significant associations as well (p>8×10^−6^) and none of the top five genes in these analyses have globally significant GWAS associations reported. For ER+ breast cancer, the burden of rare SNPs in gene *FGFBP3* was nominally globally associated (p = 6×10^−7^) although follow-up using exact logistic regression gave a larger p-value (1.0×10^−4^). This gene included five rare SNPs and no reports of any GWAS associations for SNPs near this gene are found in the GWAS catalog. When examining ER- breast cancer, the burden of variants in *MMAB* remained one of the strongest associations (p = 2.0×10^−5^). The burden of coding SNPs (all of which were rare) in *EGR2* was the leading association in the ER- analysis with a p-value from the score test of 1.2×10^−11^. A variant upstream of *EGR2* has been associated in a GWAS of Ewings sarcoma [16]. Rare variant burdens also met our criteria for global significance for *CNR1* (p = 1.7×10^−10^), *FKSG83* (p = 1.5×10^−8^), *GATM* (p = 4.8×10^−7^), and *ACSBG1* (p = 5.3×10^−7^). Again as for the single SNP results for ER- disease, these p-values were found to be overly liberal compared to an exact test (the smallest exact logistic regression p-value was 2.8×10^−5^ for *ACSBG1*)

**Table 3:** The Most Significant Associations of Each Gene's Burden of Coding Variants with Breast Cancer Risk.

	Gene	Chr	# of SNPs	OR	P
**Overall breast cancer, functional SNPs**					
	*MMAB*	12	8	1.14	0.0000497
	*SLC16A6*	17	6	1.10	0.0000541
	*INS-IGF2*	11	4	0.88	0.000124
	*ST3GAL3*	1	9	1.14	0.00016
	*SPDEF*	6	14	1.10	0.000162
**Overall breast cancer, rare SNPs**					
	*FGFBP3*	10	5	26.6	0.00000871
	*LDLRAD1*	1	10	1.63	0.0000209
	*NAALADL1*	11	36	0.54	0.000147
	*UCHL1*	4	2	11.17	0.000162
	*TXN2*	22	2	3.03	0.000182
**ER+ breast cancer, functional SNPs**					
	*SNTN*	3	3	1.76	0.0000325
	*TXN2*	22	2	3.74	0.0000373
	*SPATA16*	3	20	0.93	0.0000505
	*APOC3*	11	3	3.26	0.000257
	*APOC4*	19	6	0.85	0.000266
**ER+ breast cancer, rare SNPs**					
	*FGFBP3*	10	5	35.35	0.000000621
	*LTBP4*	19	33	0.53	0.0000199
	*TXN2*	22	2	3.74	0.0000373
	*OR6C65*	12	4	2.44	0.0000871
	*PRC1*	15	13	2.02	0.000202
**ER- breast cancer, functional SNPs**					
	*EGR2*	10	4	32.27	0.0000000000124
	*CNR1*	6	2	36.51	0.000000000168
	*MMAB*	12	8	1.37	0.0000204
	*ATP6V1H*	8	11	3.24	0.0000209
	*MRPL20*	1	3	5.65	0.0000636
**ER- breast cancer, rare SNPs**					
	*EGR2*	10	4	32.27	0.0000000000124
	*CNR1*	6	2	36.51	0.000000000168
	*FKSG83*	6	5	4.40	0.0000000146
	*GATM*	15	6	16.18	0.000000483
	*ACSBG1*	15	11	2.63	0.000000533

### Prostate Cancer Single SNP Associations

For overall prostate cancer (4,376 cases and 7,545 controls) none of the single SNP associations with prostate cancer met the Bonferroni adjustment for multiple comparison testing (nominal p<3.9×10^−7^). The top two associations found for prostate cancer were for rare NS variants in *F13A1* (rs140712764, *Val170Ile*, OR = 28.0, p = 9.1×10^−7^) and *ANXA4* (rs146778617, *Val315Phe*,OR = 4.52, p = 6.0×10^−6^), Table 4, see also Table S2. Gene *F13A1* is a coagulant factor gene not obviously related to prostate cancer etiology. *ANXA4* encodes a protein that has been discussed as a possible marker for gastric cancer [17]. Of note, the third most significant association was for a common NS variant in *GPRC6A* (rs2274911, *Pro91Ser*, OR = 0.88, P = 1.3×10^−5^). This gene is nearby to *RFX6*, which harbors an intronic variant (rs339331) that has been reported in a GWAS of prostate cancer in Japanese men [18]. The SNP rs2274911 is common in all populations (MAFs of 24–43%) (Table 4) and the protective effect of the minor allele was generally consistent in each group (OR = 0.78 to 0.95, over the five groups). This NS variant is correlated with the known intronic variant (rs339331, which is included on the Illumina HumanExome array) in all populations (r^2^ between 0.74 and 0.98) ; in conditional analyses neither of these two SNPs remained significant after the other was forced into the model (P>0.2); thus these two variants are probably capturing the same signal, with the NS SNP in *GPRC6A* a potentially plausible susceptibility variant. The top 10 ranked associations (Table 4) were all NS variants and 4 were common with a MAF>10% in all ethnic groups.

**Table 4:** The Most Significant Associations of Single Coding Variants with Prostate Cancer Risk.

All Cases (n = 4,376) vs Controls (n = 7,545)													
SNP ID^a^	Chr	Position^b^	rs#	A1/A2^c^	Type	Gene	OR^d^	P	AAMAF^e^	NHMAF	JAMAF	LAMAF	EAMAF
exm514211	6	6266854	rs140712764	T/C	*Val170Ile*	*F13A1*	28.007	9.1E-07	0.000233	0	0	0	0
exm199465	2	70052624	rs146778617	T/G	*Val315Phe*	*ANXA4*	4.523	6.0E-06	0.002563	0	0	0	0
exm574153	6	117130704	rs2274911	G/A	*Pro91Ser*	*GPRC6A*	0.875	1.3E-05	0.2379	0.2717	0.4332	0.2657	0.2542
exm68152	1	70896038	rs145785987	C/T	*Cys229Arg*	*CTH*	9.011	3.1E-05	0.000699	0	0	0	0
exm971959	11	134128968	NA	G/A	*SER186ASN*	*ACAD8*	>999.999	3.2E-05	0	0	0	0	0
exm1105738	14	61180657	rs3742636	T/G	*His605Pro*	*SIX4*	1.125	4.0E-05	0.4256	0.2742	0.4688	0.2479	0.2912
exm1474666	19	43414890	rs116433230	A/C	*Gly183Val*	*PSG6*	0.223	4.3E-05	0.01235	0	0	0.001536	0
exm506442	5	176637576	rs28932178	C/T	*Ser457Pro*	*NSD1*	0.871	6.1E-05	0.1051	0.4243	0.5249	0.217	0.1507
exm1507288	19	55527081	rs2304167	C/T	*Ala249Thr*	*GP6*	0.879	7.0E-05	0.4653	0.2903	0.2239	0.1889	0.1819
exm1478994	19	45296806	rs3208856	T/C	*His359Tyr*	*CBLC*	0.687	7.3E-05	0.04613	0.008685	0.000497	0.01882	0.04667

Advanced Cases (n = 499) vs Controls (n = 7,545)													
SNP ID^a^	Chr	Position^b^	rs#	A1/A2^c^	Type	Gene	OR^d^	P	AAMAF^e^	NHMAF	JAMAF	LAMAF	EAMAF
exm280349	2	239049718	NA	A/G	*Arg108His*	*KLHL30*	13.991	1.7E-09	0	0	0	0	0.002081
exm1488544	19	49376683	rs45533432	G/A	*Arg65Gly*	*PPP1R15A*	4.677	1.2E-08	0.002097	0.002481	0	0.00384	0.009512
exm643590	7	100634145	rs143984295	A/G	*Ala101Thr*	*MUC12*	14.425	1.5E-08	0	0	0	0.000384	0.001486
exm701486	8	55540418	rs114797722	C/G	*Ala1326Pro*	*RP1*	13.409	2.0E-08	0.001631	0	0	0	0
exm782688	9	130224593	rs150292099	G/A	*VAL157ILE*	*LRSAM1*	10.488	3.5E-07	0.002097	0	0	0	0
exm2275251	17	58235051	rs185658468	T/A	SP	*CA4*	7.137	5.1E-07	0	0.002481	0.003976	0	0
exm1321007	17	39520119	rs150620728	T/C	*Arg395His*	*KRT33B*	7.485	5.8E-07	0.002816	0	0	0.001152	0
exm942022	11	75439894	rs141331999	G/A	*Asn237Ser*	*MOGAT2*	8.489	9.4E-07	0.000932	0	0	0.000384	0.001784
exm594160	6	167343185	rs35716361	T/G	*Ser221Tyr*	*RNASET2*	8.129	1.1E-06	0.00303	0	0	0	0
exm1607984	22	38483189	NA	G/A	*Ser401Pro*	*BAIAP2L2*	6.084	2.4E-06	0.001865	0	0.001244	0.001923	0.0002973

Non-Advanced cases (n = 3,666) vs Controls (n = 7,545)													
SNP ID^a^	Chr	Position^b^	rs#	A1/A2^c^	Type	Gene	OR^d^	P	AAMAF^e^	NHMAF	JAMAF	LAMAF	EAMAF
exm514211	6	6266854	rs140712764	T/C	*Val170Ile*	F13A1	28.366	8.3E-07	0.000233	0	0	0	0
exm1228070	16	25255366	rs61746620	A/G	*Ala574Val*	ZKSCAN2	13.396	1.3E-05	0.0002331	0	0.0002485	0	0
exm199465	2	70052624	rs146778617	T/G	*Val315Phe*	ANXA4	4.275	3.4E-05	0.002563	0	0	0	0
exm574153	6	117130704	rs2274911	G/A	*Pro91Ser*	GPRC6A	0.876	4.1E-05	0.2379	0.2717	0.4332	0.2657	0.2542
exm1311040	17	32688826	rs138527286	C/T	*Ile56Val*	CCL1	2.343	4.1E-05	0.01072	0	0	0	0
exm68152	1	70896038	rs145785987	C/T	*Cys229Arg*	CTH	8.761	6.3E-05	0.000699	0	0	0	0
exm1105738	14	61180657	rs3742636	T/G	*His605Pro*	SIX4	1.127	8.6E-05	0.4256	0.2742	0.4688	0.2479	0.2912
exm392074	4	22390167	rs9002	T/C	*Val1043Met*	GPR125	1.139	1.2E-04	0.2823	0.134	0.1886	0.2342	0.202
exm1419304	19	8564474	rs4239541	T/G	*Pro73Gln*	PRAM1	1.133	1.5E-04	0.6842	0.1563	0.1499	0.3263	0.2701
exm854616	10	106025864	rs116993524	T/C	*Thr163Ile*	GSTO1	3.756	1.6E-04	0.000932	0	0	0	0.002378

a SNP ID from db135.

b Position based on GRCh37.

c A1 is minor allele based on the entire multiethnic sample and the tested allele, A2 is the reference allele.

d Odds ratio per allele based on the pooled analysis adjusted for age and the first 10 principle components.

e MAF is minor allele frequency in controls.

AA, African Americans; NH, Native Hawaiians; JA, Japanese Americans; LA, Latinos; EA, European Americans; SP, splice-site variant.

When restricted to advanced cases (n = 499), similarly as for ER- breast cancer, many associations with very rare SNPs were nominally significant using the score test (69 total for SNPs with less than 10 minor alleles observed) but the p-values failed to stand up to further investigation using exact logistic regression (with p-values all <3×10^−5^). In order to reduce discussion of a large number of likely false positive tests we considered in subtype (advanced/nonadvanced) analyses only SNPs with at least 10 minor alleles seen over all cases and controls used in the analysis. Of the remaining SNPs we found that four NS SNPs with at least 10 minor alleles present were nominally significant using the score test criteria (Table 4, Table S4). These included NS variants in *KLHL30* (exm280349, *Arg108His*, OR = 13.9, p = 1.7×10^−9^), *PPP1R15A* (rs45533432, *Arg65Gly*: OR = 4.67, p = 1.2×10^−8^), *MUC12* (rs143984295, *Ala101Thr* , OR = 14.4, p = 1.5×10^−8^) and *RP1* (rs114797722, *Ala1326Pro*, OR = 13.4 p = 2×10^−8^). These SNPs were all quite rare in the four largest populations (0.1%–1%). P-values from exact logistic regression for these SNPs were again less significant with p-values between 1.4×10^−6^ and 4.6×10^−4^).

For non-advanced disease (n = 3,666 cases), the strongest associations were with the same SNPs as overall prostate cancer (rs140712764 in *F13A1*, rs146778617 in *ANXA4*, rs2274911 in *GPRC6A*) and also with rs61746620 in *ZKSCAN2* (*Ala574Val*, OR = 13.4, p = 1.3×10^−5^), although none of these were significant at our Bonferroni criteria.

#### Ethnic-specific analyses

No SNPs were significantly associated with overall prostate cancer in ethnic specific analysis (Table S3).

### Prostate Cancer Gene Burden Analysis

None of the gene burden analyses were significant for overall prostate cancer after correcting for multiple comparisons (p<3×10^−6^) either when including common coding variants or when restricting the results to SNPs with frequency ≤1% (Table 5, Table S5). When the analysis was restricted to advanced prostate cancer, four gene burdens (for *SAMD1*, *FOXF2*, *NOL4* and *CPA3*) were significant using the score test but not by exact logistic regression (p = 2.5×10^−3^ , 3.3×10^−3^ , 5.0×10^−3^ and 3.4×10^−6^ respectively). No notable findings were observed when only localized prostate cancer was assessed.

**Table 5:** The Most Significant Associations of Gene Burden of Coding Variants with Prostate Cancer Risk.

	Gene	Chr	# of SNPs	OR	P
**Overall prostate cancer, functional SNPs**					
	*C6orf165*	6	26	0.86	0.00000573
	*MANSC1*	12	9	0.86	0.0000611
	*GP6*	19	21	0.96	0.0000642
	*SERPING1*	11	7	1.51	0.0000963
	*SIX4*	14	13	1.10	0.00012
**Overall prostate cancer, rare SNPs**					
	*C17orf53*	17	24	0.44	0.0000533
	*WDR54*	2	8	1.94	0.000112
	*TYW1B*	7	5	1.55	0.000138
	*FGFBP3*	10	5	18.04	0.000151
	*SERPING1*	11	7	3.13	0.000188
**Advanced prostate cancer, functional SNPs**					
	*SAMD1*	19	3	26.03	0.000000122
	*FOXF2*	6	2	42.26	0.000000513
	*NOL4*	18	2	17.53	0.00000213
	*IDI1*	10	3	13.41	0.0000124
	*CYP11B1*	8	5	>999	0.0000207
**Advanced prostate cancer, rare SNPs**					
	*CPA3*	3	19	2.26	0.0000000697
	*SAMD1*	19	3	26.03	0.000000122
	*FOXF2*	6	2	42.26	0.000000513
	*NOL4*	18	2	17.53	0.00000213
	*IDI1*	10	3	13.41	0.0000124
**Non-advanced prostate cancer, functional SNPs**					
	*ATP6V0D2*	8	17	0.75	0.0000749
	*C6orf165*	6	26	0.87	0.0000913
	*MANSC1*	12	9	0.86	0.000108
	*GP6*	19	21	0.96	0.000113
	*GPR125*	4	37	1.11	0.000143
**Non-advanced prostate cancer, rare SNPs**					
	*C17orf53*	17	24	0.40	0.0000433
	*SEMA4B*	15	22	1.44	0.000172
	*FGFBF3*	10	5	18.06	0.000181
	*SERPING1*	11	7	3.17	0.000266
	*WDR54*	2	8	1.88	0.000454

### Analyses at Known Risk Loci for Breast and Prostate Cancer

#### GWAS loci

Tables S6 and S7 give results for SNP associations for genes located at known breast and prostate cancer susceptibility regions revealed through GWAS (e.g. regions harboring globally significant associations) as of the time of this report (73 significant associations for breast cancer and 89 for prostate cancer [19], [20]). For each region, we list the genes having one or more genotyped coding variants that lie within 500 kb of the known GWAS SNP and summarize associations (smallest p-value) with coding variants in those genes and with the burden of coding variants (all SNPs and rare SNPs). For breast cancer, we observed limited evidence of associations with rare coding variants in genes proximal to GWAS signals, with 9 genes (*PTPN22*, *PTPN7*, *MDM4*, *CASP8*, *SLC6A18*, *FOXF2*, *CTSW*, *CCDC88C*, *ZNF404*) having SNPs or gene burdens achieving p-values of p<0.05 (Table S6) after correcting for either the number of nearby (+/−100 kb) SNPs (single SNP analyses) or genes (gene burden) for each GWAS index association. Of SNPs in linkage disequilibrium (LD), r^2^>0.3 (in Europeans in 1000 Genomes), with GWAS hits we identified NS SNPs in 2 genes (*STXBP4* and *ZNF404*) which were correlated with 2 index GWAS SNPs (rs6504950 and rs3760982) and associated weakly at p<0.05.

For prostate cancer, the most significant GWAS-related association, as described above, was with rs2274911 (*Pro91Ser*) in *GPRC6A.* The next most significant finding was with rs16836525 (*Val125Met*) in *PMVK* at 1q21 (p = 3.0×10^−4^). This SNP was only common in African Americans (20% frequency; ≤1% in the other populations). An additional eight nearby genes had SNPs with corrected p-values between 0.001 and 0.05:, *ITGA6*, *VGLL3*, *TECPR1*, *TPCN2*, *FAM83F*, *PBXIP1*, *FARP2* and *TTLL12* (Table S7). Seven SNPs were correlated with a GWAS index SNP at r^2^≥0.3 in the 200 kb window and significant at p<0.05 (*SLC2A4RG*, *PDLIM5*, *RNMTL1*, *KLK3*, *MLPH*, *RTEL1* as well as *GPRC6A*).

Given the modest effects noted with the initial GWAS signals as well as observed with these correlated coding SNPs (OR per allele of ∼1.1; Table S6 and S7), and the lack of strong signals noted for the index signals across populations [21] conditional analyses will be needed in much larger samples of the GWAS population (mainly European ancestry) to determine whether these coding SNPs are the biologically functional alleles underlying the GWAS signal. (Our ability to perform informative conditional analysis here is further hampered by the fact that only a minority of the index GWAS hits are included on the Illumina array).

#### Extended associations

Because of the interest in the possibility that rare coding variants with large effect sizes (OR>1.5 or higher) may underlie GWAS signals and since LD with rare SNPs can extend much further than with common SNPs, we report in Table S6 and S7 the strongest associations for all coding variants in each gene within 500 kb of each GWAS index signal. The strongest single SNP associations with breast cancer (from 100 to 500 kb) were in *INS-IGF2* (260 kb from rs3817198 on chromosome 11, p = 1.1×10^−4^), *CCDC91* (450 kb from index signal rs10771399 on chromosome 12, p = 4.7×10^−4^), *ZFYVE26* (410 kb from rs2588809 on chromosome 14, p = 7.9×10^−4^), *C16orf46* (444 kb from rs13329835 on chromosome 16, p = 2.8×10^−4^) *UNC13A* (337 kb from rs8170, p = 5.7×10^−4^) and *NRIP1* (182 kb away from rs2823093 on chromosome 21, p = 1.3×10^−4^).

For prostate cancer the strongest such associations were with *SNED1* (412 kb from rs3771570 on chromosome 2, p = 3.5×10^−4^) and *PASK* (317 kb from the same index SNP on chromosome 2, p = 4.8×10^−4^). No other associations in this distance range had p<0.001 for overall breast or prostate cancer.

#### High-risk genes

We also examined genes implicated in family-based studies of breast or prostate cancer (Tables S6 and S7) as they are strong candidates. For breast cancer, we analyzed 11 genes and did not observe an over-representation of associations at p<0.05 in any gene (observed/tested: *ATM*, 3/62; *BRCA1*, 2/42; *BRCA2*, 3/80; *BRIP1*, 0/16; *CHEK2*, 0/8; *NBN*, 3/17; *PALB2*, 1/26; *PTEN*, 0/1; *RAD50*, 3/24; *STK11*, 1/4; *TP53*, 0/4), or any significant associations (p<0.05) from gene-based burden testing. The most significant associations in these genes (p<0.05) were noted with non-synonymous variants: rs56009889 in *ATM* (*Phe2307Leu*, OR = 4.13, p = 0.0065), rs80357090 in *BRCA1* (*Val199Ile*, OR = Inf, p = 0.018), rs1799944 in *BRCA2* (*Asn991Asp*, OR = 0.83, p = 0.0046), rs115321485 in *NBN* (*Lys628Glu*, OR = 0.41, p = 0.0067), rs2230017 in *RAD50* (*Ile291Thr*, OR = 0.44, 0.0069) and rs138789658 in *PALB2* (*Lys18Arg*, OR = 1.69, p = 0.03).

For prostate cancer, we analyzed 5 genes and did not observe an over-representation of SNP associations at p<0.05 (observed/tested: *BRCA2*, 2/83; *ELAC2*, 1/9, *HOXB13*, 0/2; *MSR1*, 1/22; *RNASEL*, 2/21). However, we did observe suggestive evidence of associations with burden testing of rare (MAF<0.01) SNPs in *ELAC2* (OR = 1.67, p = 0.03) and in *RNASEL* (OR = 1.26, p = 0.02). The most significant associations included a very rare NS variant in *ELAC2* that was mainly observed in African Americans(rs149544601, *Ile356Val*, MAF = 6.6×10^−5^; OR = 14.0, p = 0.0014), and a nonsense SNP (rs74315364, *Glu265Ter*) and NS variant (rs151296858, *Gly59Ser*) in *RNASEL* (both with OR = 2.51, p = 0.012) that were observed in the same individuals. We did not observe significant associations with any of the reported risk variants in these genes (*Ala541Thr*, *Ser217Leu* in *ELAC2*; *Ser41Thr*, *Asp174Tyr*, *Pro275Ala*, *Arg293Ter* in *MSR1*, or *Arg462Gln*, *Glu541Asp* in *RNASEL*, Table S7; the recently reported *HOXB13* variant, *Gly84Glu* [22], was not included on the array).

### Other Phenotypes and Traits

We also examined additional cancer-related traits: body mass index (BMI), alcohol intake, as well as circulating PSA levels (Table S8). A number of NS variants have already been strongly associated with many of these traits, such as rs671 (*Glu504Lys*) in *ALDH2* with alcohol intake [23], rs17632542 [*Ile179Thr*] in *KLK3* and circulating PSA levels [24], [25] and rs198977 [*Arg250Trp*] in *KLK2* and the ratio of free to total PSA [26]. For each trait, the 10 most associated variants on the array (including non-functional SNPs, i.e. GWAS SNPs) are provided in Table S9. We also observed a number of suggestive associations at p<3.9×10^−7^ with rare coding variants in some genes that are biologically plausible for each trait. Three variants were strongly associated with blood PSA levels (chr19: Hg19 position: 4552446, *Thr326Met*, *SEMA6B*, 0.1% MAF in African Americans and monomorphic in all of the other populations, beta = 3.8, p = 3.8×10^−9^; rs17632542, *Ile136Thr*, beta = −0.4588, p = 1.0×10^−8^ MAF.06 in European Americans; rs148595483, *Asn322Lys*, *CCDC78*, 0–0.1% MAF across populations, beta = −2.9, p = 2.4×10^−8^). We also found a number of significant associations with very rare NS variants that were observed in 2–7 individuals and BMI (rs146199292, *Asn31Lys*, *OSBPL11*, beta = 19.9 p = 1.2×10^−10^; rs149954327, *Leu458Val*, *STON1-GTF2A1L*, beta = 15.2 p = 1.5×10^−9^; rs146922831, *Lys608Asn*, *LRGUK*, beta = 9.2, p = 3.0×10^−8^). The variants were very rare in African Americans with frequencies <0.09% and monomorphic in all of the other populations except for rs146199292 in Latinos (0.02%). Variations in these genes have been reported in association with conditions related with BMI, including cardiovascular risk factors, type 2 diabetes and polycystic ovarian syndrome [27], [28], [29]. The carriers of these rare alleles were clustered at the extreme high end of the BMI distribution. All these potentially novel associations will need further follow-up.

This paper presents an initial investigation of the role of coding variation in the genetics of breast and prostate cancer. Our initial analysis fails to find strong evidence for the hypothesis that relatively rare coding variation is highly determinative of breast or prostate cancer risk either overall or by subtype. Our sample sizes in each racial/ethnic group were each relatively small (roughly 1,000 cases and 2,000 controls in the largest groups) however these sample sizes are large enough to detect risk alleles with moderate to large effects (odds ratios of 3–13) appearing in quite low frequency (0.1–1%) and to examine whether such coding variation underlie (by so-called synthetic association [30]) many GWAS associations. While caution is advised in interpreting our results, especially for other than European racial/ethnic groups (since the array utilized was predominantly based upon sequence information for Europeans and is not expected to cover other groups equally well), it appears that future studies to understand the relationship between rare coding variation and breast and prostate cancer risk will likely require the very large sample sizes needed to target much less penetrant alleles.

Our analyses consisted of both single variant analysis and simple gene burden analyses. The gene burden analyses consisted of summing the minor alleles of coding variants including either all coding variants regardless of their frequency, or only those variants with MAF <1% in our overall sample. While this gene-burden test assumes implicitly that all coding variants have the same direction of effect, this is reasonable given that the power of detecting rare protective alleles in a case-control study such as this one (where controls can be regarded as representative of the population) is much less than the power to detect rare risk alleles. The rare variant sum therefore is not very sensitive to the presence of rare protective alleles in a gene.

One association for breast cancer, a single SNP in *LDLRAD1*, appeared to pass our established level of global significance (p<3.9×10^−7^) when all cases were examined. No associations (either single SNPs or gene burdens) were globally significant for overall prostate cancer. Subset analyses, by ER status for breast cancer or advanced/non-advanced for prostate cancer generally failed to show believable associations. While the score test gave many “globally significant” associations these apparently reflected excess type I error of this test when both the number of cases is small compared to the number of controls and when the SNPs were rare. This breakdown in reliability is similar to that seen for the uncorrected Pearson chi-square test (a special case of the score test when no covariates are present), which is well-known to have poor control of type I error when the expected number of cases is very small for a cell. Following-up such associations with exact logistic regression implemented in SAS (Cary, NC) provided larger p-values not globally significant using our criteria.

Nevertheless a number of suggestive findings were observed that are worthy of further attempts at replication: The splice site variant rs145889899 in *LDLRAD1* (our top finding for overall breast cancer) is found in low frequency (<1%) in African American controls (higher of course in cases since this is nominally a risk variant), and only seen among cases in the other groups. No associations with any disease or phenotype have to date been reported for this gene. Among the other genes highlighted in Table 2 or Table 3, associations have been reported for SNPs in *SLC19A1* and *CASP5* for renal cancer [31], [32]; *BAZ2A* has been reported to be up-regulated in CLL patients [33]. Also notable is a strong link between SNPs in *EGR2* (ER- association) and risk of Ewing's sarcoma [16].

For prostate cancer (all cases) the third strongest association result was for a common NS coding variant (rs2274911) in *GPRC6A* that is in very high LD with the known intronic GWAS variant rs339331. In our data the NS variant was slightly more associated (Table S3) with prostate cancer risk (p = 1.3×10^−5^) than was rs339331 (p = 2.1×10^−5^). The coding SNP is arguably a more likely causal variant than the intronic SNP since expression of *GRPC6A* is substantially increased in prostate cancer cell lines, and mice deficient in *GRPC6A* show retarded prostate cancer progression [34]. In addition, *GRPC6A* deficiency in mice also attenuates the rapid signaling responses to testosterone, an androgen that is critical for initiation and progression of prostate cancer [35].

Other suggestive findings for prostate cancer include SNPs in a variety of genes such as *F13A1* expression of which has been associated with bone metastasis in prostate cancer [36], *ANXA4* which is up-regulated in gastric and other cancers [17], *NSD1* where cryptic translocations may be involved in AML occurrence [37] and *MUC12*, expression of which has been reported to be a prognostic marker in colon cancer [38]. The burden of rare SNPs in *FGFBP6* (one of the stronger association seen for breast cancer) was also among the top associations for overall prostate cancer (Table 5, p = 1.5×10^−4^).

We evaluated also associations in regions surrounding known (GWAS) risk alleles as a partial fine-mapping exercise; we specifically focused upon (1) coding alleles reported to be in high LD (in Europeans using 1000 Genomes data) with the index marker, and (2) other (generally less common) coding alleles within 500 kb of the GWAS alleles, that might show associations that could underlie (by synthetic association [30]) GWAS associations. A number of GWAS risk alleles are in reasonable LD (r^2^>0.3) with coding SNPs on the array and several of the latter show nominal associations (p<0.05) with breast cancer risk including SNPs in *STXBP4*, *ZNF45*, and *ZNF404* which are all worth evaluating as candidate loci potentially explaining the index GWAS associations. For prostate cancer, a similar observation is made most notably for *GPRC6A* but also for *MLPH* (GWAS index = rs7584330, chromosome 2, p = 0.003), *PDLIM5* (rs12500426, chromosome 4, p = 0.019), *RNMTL1* (rs684232, chromosome 17, p = 0.024), *KLK3* (rs2735839, chromosome 19, p = 0.0046), and *RTEL1* (rs6062509, chromosome 20, p = 0.001). Previous reports [24], [39] have highlighted the NS SNP rs17632542 in *KLK3* as highly associated with PSA level and a highly significant risk variant in fine-mapping of the locus near rs2735839 [39]; while no report for prostate cancer exists for coding SNPs in *RTEL1*, another NS SNP, rs3208008, in *RTEL1* has been found to be associated with glioma risk [40].

Other coding SNPs that could include causal variants producing synthetic associations (associations of rare with common SNPs of high penetrance) include SNPs in genes *INS-IGF2*, *ZFYVE26*, *C16orf46*, *UNC13A*, *NRIP1* and *CCDC91* for breast cancer and SNPs in *SNED1* and *PASK* for prostate cancer. These do not have high r^2^ with the GWAS variants as they are mostly rare (and are >100 kb away from the index signal) but their nominally strong associations (p-values<1×10^−3^) might possibly be indicative of signals extending for many thousands of base pairs, although it will take much larger studies to verify or refute this.

We found little evidence that the NS, SP, or nonsense variants captured by the HumanExome SNP array that fall within known or suspected high risk genes for breast or prostate cancer are meaningfully associated with either cancer. The Illumina array does not directly interrogate the rare, high-risk mutations, such as frameshift mutations in *BRCA1* or *BRCA2* (e.g. c.68_69delAG) [41], as very few indels are included on this array (just 136 were examined here). The inability to address frameshift mutations either within known risk genes or more widely is a limitation of this report. Other limitations include the focus on Europeans in the development of the array (as seems to be particularly reflected in the relatively small fraction of SNPs found to be polymorphic in Japanese Americans), and the loss of some targeted SNPs in the manufacturing process and in our QC procedures. In addition, this technology (unlike exome sequencing) cannot address the role of either private variation or of variants too rare to have been reliably identified during the discovery phase of the development of the array.

Genotyping cases and controls from our prospective cohort allowed us an opportunity to examine other cancer-related phenotypes and traits for which data and specimens had been collected prior to breast or prostate cancer diagnosis. While two of these endpoints (BMI, alcohol) were based on self-report, we were able to strongly replicate a number of known associations such as rs671 in *ALDH2* with alcohol intake which is proof of principle that the exome array has the potential to reveal biologically relevant coding variants. Apparently novel findings for PSA, BMI, and alcohol consumption will need to be replicated in large-scale exome association analyses; hopefully making the results from these preliminary analyses in a multiethnic population broadly available will contribute to novel discoveries and further understanding the genetic basis of these traits.

In order for rare variants to play an important role in explaining missing heritability [42] even in composite they must have effects that are larger in magnitude than those observed for common SNPs. Roughly speaking, for a given allele the contribution to additive heritability (under a liability model for example [43]) is proportional to 2b^2^p(1-p) where b is the log odds ratio (OR) and p is the frequency for that allele. Under simplifying assumptions (such as limited selection and constant population sizes) population genetics theory [44] indicates that there should be approximately as many variants “moderately rare” with frequency in the range 0.1 to 1% as there are the common variants in the range 5 to 50% that have been the targets of GWAS studies to date. However, in order that variants in the frequency range from 0.1 to 1% have the same composite effects on risk as do those in the frequency range from 5 to 50% then the magnitude of effect sizes must be considerably larger than for the common variants; if ORs for common variants lie in the range from 1.1 to 1.3 then ORs in the range from 2 to 6 are needed for the rare and common alleles to have similarly sized roles in disease susceptibility (assuming that the same fraction of all rare alleles are risk variants as for common alleles). Moreover, under the hypothesis that the coding regions of the genome (∼1% of the total genome) by themselves play an profound role in disease susceptibility these ORs would likely need to be skewed even higher – i.e. if rarer variation in 1% of the genome was to play as much a role as does common variation over the entire genome then the existence of ORs above 10 or even greater for such variation may arguably be a necessary consequence.

Realistically our study only begins the assessment of whether a range of effects for “moderately rare” coding variants is possible: the detectable ORs in this study range from approximately 3 to 13 for alleles with frequency 1 to 0.1%, respectively. While these are large ORs the above argument indicates that such effect sizes are not unreasonable if rarer protein coding variation plays a similar role in the heritability of risk as does common variation genome-wide. Our failure to find such ORs for the rarer alleles may be providing evidence against coding variation having a predominant role in breast and prostate cancer heritability and risk (outside of high risk families).

In summary, the analyses and methods described here do not support NS variants on the current exome chip as conveying moderate to high risk for breast and prostate cancer. While some suggestive findings are noted it is likely that very large sample sizes of the order that can be only developed through collaborative efforts such as those now engaged in the NCI GAME-ON post-GWAS meta-analysis of common variants, will be required in order to further the understanding of the role of rare NS and other coding variation in disease genetics. Exome sequencing of high-risk families will continue to be important to reveal biologically relevant coding variants for these cancers, both for insertion/deletion variants that were not covered by the current array, and to capture rarer variation (including private variants) that cannot be captured except by sequencing.

## Materials and Methods

### Ethics Statement

This work has been performed according to relevant national and international guidelines. Written consent was obtained at the time of DNA sample collection. The Institutional Review Boards at the University of Southern California and University of Hawaii approved of the study protocol.

### Study Population

The MEC consists of more than 215,000 men and women in California and Hawaii aged 45–75 at recruitment, and comprises mainly five self-reported racial/ethnic populations: African Americans, Japanese, Latinos, Native Hawaiians, and European Americans [45]. Between 1993 and 1996, adults enrolled in the study by completing a 26-page mailed questionnaire asking detailed information about demographic factors, personal behaviors, and prior medical conditions. Potential participants were identified through driver's license files from Departments of Motor Vehicles, voter registration lists, and Health Care Financing Administration data files. Incident breast and prostate cancer, as well as stage and hormone receptor status was identified by linkage of the cohort to the Surveillance, Epidemiology, and End Results cancer registries covering Hawaii and California. Between 1995 and 2006, blood specimens were collected prospectively from ∼67,000 participants for genetic and biomarker analyses. Currently, the breast cancer case-control study nested in the MEC includes 3,141 women diagnosed with invasive breast cancer and 3,721 frequency-matched controls without breast cancer, matched by race/ethnicity and age (in 5-year age categories). The case-control study of prostate cancer includes 4,675 men diagnosed with incident prostate cancer and 4,300 male controls without prostate cancer. The Institutional Review Boards at the University of Southern California and University of Hawaii approved of the study protocol.

### Genotyping and Quality Control

Genotyping of the Illumina Human Exome BeadChip (n = 247,895 SNPs) was conducted at the USC Genomics Core Laboratory.

DNA extraction of buffy coat fractions was conducted using the Qiagen protocol. Cases and controls were randomly placed across ethnic-specific plates for each cancer type. All samples had DNA concentrations >10 ng/ul. Initial genotype definitions were based on auto-clustering 6,404 samples across all populations which had call rate >0.99 (African American 1883, Japanese American 1823, Latino 1008, European American 1690) using the GenomeStudio software (V2011.1). Following genotype calling on all samples (>16,000), manual inspection was conducted of the following SNPs: 1) SNPs with call rate <0.98 (n = 3,317), 2) monomorphic SNPs with call rate <1 (n = ∼15,000), 3) SNPs with minor allele frequency between 0 and 0.001 and call rate <1 (n = ∼31,500), 4) SNPs with >1 replicate error based on sample duplicates (∼1,000, discussed below), 5) SNPs with apparent differences in minor alleles frequencies >15% across ethnic-specific 96 sample plates (n = 798), or other evidence of batch/plate effects on allele frequency (n = 18,188), 6) all mitochondrial SNPs and all SNPs on the X and Y chromosomes (n = 5,574), and 7) autosomal SNPs out of Hardy-Weinberg Equilibrium in more than one ethnic group with p value<0.001 and at least one ethnic group with p value<0.00001 (n = 827). During the inspections we in total inspected cluster plots for approximately 70,000 SNPs (counting overlapping SNPs in the categories above) and genotypes were manually edited for 27,506 SNPs.

Of the 15,837 samples described above genotyping was successful with call rates ≥98% for 15,573 samples; of these we removed 17 samples for which reported sex conflicted with assessment of X chromosome heterozygosity, and 651 samples based on relatedness. Relatedness was determined using the IBD calculation in plink [46], and we removed one of each estimated MZ twin, sibling, parent-offspring, half sibling, or first cousin pairs. In the analysis, we also removed SNPs with <98% call rates (n = 2,531). To assess genotyping reproducibility we included 338 replicate samples which passed genotyping QC; among these samples the concordance rate of heterozygote calls, number concordant/(number concordant+number discordant), was 99.6% or greater for all replicate samples (average 99.99%). The final analysis dataset included 245,339 SNPs genotyped on 2,984 breast cancer cases and 3,568 controls, and 4,376 prostate cancer cases and 3,977 controls.

### Statistical Analysis

We relied on documentation files obtained from the University of Michigan posted on ftp://share.sph.umich.edu/exomeChip/IlluminaDesigns/ for the assessment of SNP type (i.e. NS, SP), and the amino acid affected. The array also includes SNPs that do not code for protein changes including synonymous SNPs, and other intergenic SNPs including ancestry informative markers, and GWAS identified risk SNPs for a number of diseases and outcomes. All SNPs were analyzed and their results shown in Tables S1, S2, S3, S4, S5, S6, S7, S8, S9. However our primary analysis focused on the 191,032 putative functional variants in the following categories (NS, SP and stop gain or loss) that passed quality control procedures discussed above.

We estimated principal components in the entire sample using EIGENSTRAT [47] based on 2,887 autosomal ancestry informative markers on the array. We adjusted for the top 10 principal components in all analyses.

#### Association testing of single markers

For all analyses except those of the X and Y chromosomes all controls (men and women combined) were utilized in the analysis of each cancer in order to increase statistical power. Only controls of the same sex were used to analyze X or Y chromosome variants. Analyses were performed overall and within each racial/ethnic group. For each genotyped SNP, odds ratios (OR) and 95% confidence intervals (95% CI) were estimated using unconditional logistic regression of case/control status adjusting for age at diagnosis (cases) or blood draw (controls), and reported race/ethnicity in the overall analyses, and the first 10 eigenvectors in both overall and ethnic-specific analyses. For each SNP, we tested for allele dosage effects through a 1 d.f. score chi-square trend test. When exposures are rare but with very strong effects the score test can be more powerful than the usual Wald test for reasons described in Hauck and Donner [48]. However, we found the score test to be overly liberal when both the exposures are rare and when (as in analysis of advanced prostate and ER- breast cancer) the number of cases in a given analysis is small compared to the number of controls. Therefore we followed up any apparently globally significant findings found with the score test by rerunning that analysis using the exact logistic regression procedure implemented in SAS (Cary NC); when using the exact test we dropped the eigenvectors and age from the analysis and only used reported race/ethnicity as an adjustment variable. The presentation of results is focused on putative functional exonic SNPs (i.e. NS, SP and stop gain or loss); the most significant results for all SNPs (including non-functional SNPs) are provided in Tables S1, S2, S3, S4.

#### Gene burden scores

For each gene listed in the annotation files we conducted a simple gene-specific burden test summing the number of minor alleles of each putative functional SNP carried by an individual. These summation variables were then used as the genetic variable in logistic regression models of case-control status after adjusting for age, reported race/ethnicity, and the first 10 eigenvectors above. We performed the gene burden analyses twice, once using all putative functional SNPs and again using only those variants with MAF<1% in the total sample. Statistical significance was again evaluated using the score test and exact logistic regression. The use of a simple gene burden analysis is over-simplified since it implicitly assumes all effects are in the same direction. It is important to remember however that the power to detect rare protective alleles is much smaller than the power to detect rare risk alleles since the former will not be over-enriched in our controls; therefore we expect that the simple sum of minor alleles, especially for rarer alleles, will not be very much diluted by rare protective effects.

For breast cancer we ran each of the above single SNP and gene burden tests separately by estrogen receptor status (+/−); for prostate cancer we ran analyses overall and separately by classification into advanced (stage>1) versus non-advanced (stage = 1) disease. For the other traits described above, we analyzed single SNPs using regression (logistic or linear) methods for binary or continuous phenotypes. The 100 most statistically significant results for each phenotype are presented in Tables S1, S2, S3, S4, S5.

#### Evaluation of the known risk loci for breast and prostate cancer

We also examined whether known risk alleles (generally intergenic or intronic) from GWAS studies of breast or prostate cancer may be reflecting an underlying signal from a nearby protein-altering variant. In these analyses for each GWAS SNP (73 for breast, 83 for prostate cancer) we initially interrogated nearby SNPs known to be or likely to be in LD with the index signal. Because LD data is not yet available for the majority of the SNPs on the HumanExome array, we expanded the associations considered to be all those within a 100 kb region on either side of the index signal, since LD between common SNPs can sometimes extend this far. In this region we highlighted in the results section and discussion, SNPs with modest signals of association (p<0.05) as well as more strongly significant SNPs. Here the common SNPs are likely to be in high LD with the (generally common) GWAS variants, and the rare SNPs could be producing synthetic associations. We then relaxed this 200 kb region to 1 mb (500 kb on either side of the index signal) in order to expand our examination of possible synthetic associations between rare SNPs and the index GWAS findings, since LD with rare SNPs can extend considerably further than with common SNPs.

### Power Analyses

Recognizing that many variants are only polymorphic in a few racial/ethnic groups, we give power analysis for a study with 1,000 cases and 2,000 controls (roughly the number of cases and controls in each of the four largest populations) by odds ratio (1–200) and allele frequencies ranging from 0.0001 to 0.1 (Figure S2). The Bonferroni criteria for significance in this study is calculated to be 0.05 divided by the largest number of polymorphic SNPs in any population (African Americans, ∼125,000) or roughly 3.9×10^−7^. For the gene burden analysis the Bonferroni criteria is 0.05 divided by the number of genes considered or roughly 3×10^−6^. We had 80% power to detect odds ratios of 3.3 or above for SNPs with a frequency of 0.01 and odds ratios in the range 13 or above for SNPs of frequency 0.001 in single SNP analyses. Power for the gene burden analysis depends upon the number of polymorphic SNPs in a given gene. Using a Poisson approximation (i.e. with variance assumed to be equal to the mean) a gene with 10 variants each of frequency 0.001 gives power of 80% to detect a per minor allele OR of 3.1. For genes with many more variants (100) of the same frequency detectable ORs per minor allele are 1.6 or greater. For common variants present in all ethnic groups we had much greater power to detect associations, for example we had 80% power to detect a 20% allele with an OR of 1.24 in the global analyses; for the region-specific analyses we have 80% power to detect a 20% allele with an OR of 1.17 in a region with 100 variants and 1.14 in a region with 10 variants.

## Supporting Information

Figure S1 Allele frequency of putative functional SNPs for a. All ethnicities combined; b. European American; c. African American; d. Latino; e. Japanese American; f. Native Hawaiian
(DOCX)

Figure S2 Statistical power for single SNP analyses.
(DOCX)

Table S1 One hundred most significant single SNP associations with breast cancer; over all ethnic groups (S1.1) and by ethnic group (S1.2–6).
(XLSX)

Table S2 One hundred most significant associations between single SNPs and (S2.1) ER-positive Breast cancer and (S2.2) ER-negative breast cancer.
(XLSX)

Table S3 One hundred most significant single SNP associations with prostate cancer; over all ethnic groups (S3.1) and by ethnic group (S3.2–6).
(XLSX)

Table S4 One hundred most significant associations between single SNPs and (S4.1) advanced prostate cancer and (S4.2) localized prostate cancer.
(XLSX)

Table S5 Gene burden analyses. One hundred strongest associations with (S5.1) Overall breast cancer, (S5.2) ER-positive breast cancer, (S5.3) ER-negative breast cancer, (S5.4) Overall prostate cancer, (S5.5) Advanced prostate cancer and (S5.6) Non-advanced prostate cancer.
(XLSX)

Table S6 Relationship between SNPs or genes known to be associated with breast cancer and coding SNPs on the exome array. Summary of nearest coding snps and gene burden analyses for (S6.1) GWAS associations and (S6.2) High risk genes.
(XLSX)

Table S7 Relationship between SNPs or genes known to be associated with prostate cancer and coding snps on the exome array. Summary of nearest coding snps and gene burden analyses for (S7.1) GWAS associations and (S7.2) High risk genes.
(XLSX)

Table S8 Summary statistics for other phenotypes examined: BMI, alcohol intake, and PSA.
(XLSX)

Table S9 Most significant single SNP association results for other phenotypes examined.
(XLSX)
