# Supplementary material for: Genome-Wide Testing of Putative Functional Exonic Variants in Relationship with Breast and Prostate Cancer Risk in a Multiethnic Population
Source: PLoS Genet. 2013 Mar 28;9(3):e1003419. doi: 10.1371/journal.pgen.1003419 (PMC3610631; doi:10.1371/journal.pgen.1003419)
Supplement: Figure S1 — Allele frequency of putative functional SNPs for a. All ethnicities combined; b. European American; c. African American; d. Latino; e. Japanese American; f. Native Hawaiian (DOCX) [file pgen.1003419.s001.docx]

**Supplemental Figure S1**. Allele frequency of putative functional SNPs for a. All ethnicities combined; b. European American; c. African American; d. Latino; e. Japanese American; f. Native Hawaiian
