# Supplementary figures and images for: Genome-Wide Testing of Putative Functional Exonic Variants in Relationship with Breast and Prostate Cancer Risk in a Multiethnic Population
Source: PLoS Genet. 2013 Mar 28;9(3):e1003419. doi: 10.1371/journal.pgen.1003419 (PMC3610631; doi:10.1371/journal.pgen.1003419)

**Supplemental Figure S2**. Statistical power for single SNP analyses.


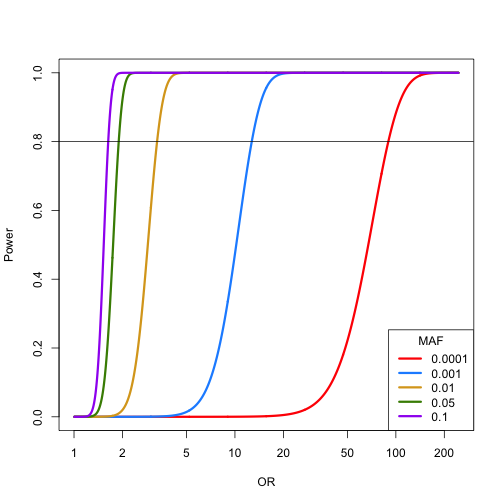

Supplement: Figure S2 — Statistical power for single SNP analyses. (DOCX) [file pgen.1003419.s002.docx]
